# Complementary and alternative therapies for generalized anxiety disorder: A protocol for systematic review and network meta-analysis

**DOI:** 10.1097/MD.0000000000032401

**Published:** 2022-12-23

**Authors:** Kai Song, Yating Wang, Li Shen, Jinwei Wang, Rong Zhang

**Affiliations:** a Department of Rehabilitation, Sichuan Vocational College of Health and Rehabilitation, Zigong, Sichuan Province, China.

**Keywords:** complementary and alternative medicine, GAD, network meta-analysis, systematic review, therapies

## Abstract

Generalized anxiety disorder (GAD) is a prevalent and impairing psychiatric disorder, which is a great threat to people’s mental health, and imposes a major burden on individuals, families and society. Complementary and alternative medicine (CAM) therapies is a potential treatment for GAD, however, the selection strategies of different CAM therapies in clinical practice is still unclear, and the purpose of this study is to evaluate the efficacy and acceptability of different CAM interventions using systematic review and network meta-analysis (NMA).

**Methods:**

Based on the strategy, the authors will retrieve a total of 9 electronic databases by January 2023. After a series of screening, the 2 researchers will use Aggregate Data Drug Information System (ADDIS) and Stata software to analyze the data extracted from randomized controlled trials (RCTs) of CAM therapies for the GAD. Finally, the evidence grade of the results will be evaluated.

**Results:**

This study will provide a reliable evidence for the selection of CAM therapies for GAD.

**Conclusion:**

The results of this study will provide references for evaluating the influence of different CAM therapies for GAD, and provide a choice basis for clinical decision-making.

## 1. Introduction

Generalized anxiety disorder (GAD) is a chronic mental disorder characterized by excessive, uncontrollable and persistent anxiety, as well as worry and fear, which usually last for at least 6 months. The term “GAD” was first used in ICD-9.^[1,2]^ DSM-V states that patients with GAD may have physical symptoms such as fatigue, restlessness, dizziness, palpitations, muscle tension, sleep disorders, etc.^[3]^ These symptoms may seriously affect the daily life of GAD patients, and may lead to functional damage in GAD patients.^[4]^ A study published in “JAMA Psychiatry” pointed out that the global lifetime total prevalence rate of GAD was 3.7%, the annual prevalence rate was 1.8%, and the monthly prevalence rate was 0.8%. In addition, the comorbidity rate of GAD was 81.9%, which increased the risk of mental and physical health of GAD patients.^[5]^ As the working ability of GAD patients decreases, with the increasing social pressure and the lack of effective anxiety management, more medical resources will be used, this will undoubtedly increase the social burden and the economic pressure of GAD patients.^[6]^ In the current clinical practice, pharmacological intervention is one of the main treatment methods of GAD, mainly include selective serotonin reuptake inhibitors and serotonin norepinephrine reuptake inhibitors.^[7]^ However, these pharmaceuticals may not be well tolerated in patients with GAD, some studies have shown that both selective serotonin reuptake inhibitors and serotonin norepinephrine reuptake inhibitors are related to the decline of efficacy at high doses.^[8,9]^ In addition, these pharmaceuticals may inevitably increase the risk of adverse effects, such as somnolence, sexual dysfunction, dry mouth, constipation, all of which affect the quality of life of patients with GAD.^[10]^ Therefore, for patients with GAD, a safer and more effective treatment strategy is urgently needed.

Complementary and alternative medical therapies includes any therapeutic practice that does not belong to the field of traditional medicine, including herbal medicine, massage therapy, acupuncture, yoga, tai chi, meditation, qigong, mindfulness relaxation exercises, etc, compared with traditional medicine, complementary and alternative medicine (CAM) therapies is generally considered as a natural and safer choice to solve common health problems.^[11]^ In recent years, the acceptance and use of CAM therapies are increasing globally. There is evidence that music therapy, yoga, meditation, acupuncture and massage may be useful auxiliary measures to treat mental health problems and help alleviate the anxiety disorder of patients.^[12]^ The research of Yang XY et al shows that compared with the control group, the acupuncture therapy aimed at reducing the anxiety of patients with GAD has certain beneficial effects.^[13]^ Yoga is a safe and widely popular therapy among people, including body postures and exercises, breathing regulation, relaxation, meditation and mindfulness exercises. Research shows that yoga therapy is an effective treatment for GAD.^[14,15]^ Systematic review research shows that herbal medicine may have the prospect of treating GAD, and herbal medicine can effectively treat GAD with fewer adverse reactions.^[16,17]^ A waiting list control study showed that mindfulness therapy for anxiety symptoms had significant clinical effects.^[18]^ Meta-analysis shows that Tai Chi intervention has beneficial effects on a series of mental health measures of different populations, including anxiety, stress and emotional disorders.^[19]^

However, the difference in efficacy between different CAM therapies is still uncertain. Different from traditional pairwise meta-analysis, Network meta-analysis (NMA) can synthesize the effect sizes of several studies that evaluate multiple interventions or treatments through indirect comparison.^[20]^ Therefore, the goal of this study is to evaluate the differences between different CAM therapies, It is expected to provide reference for clinical decision-making.

## 2. Methods

### 2.1. Protocol and registration

This protocol follows the Preferred Reporting Items for Systematic Reviews and Meta-Analysis Protocols (PRISMA-P) guideline.^[21]^ The NMA protocol has been registered on the International Prospective Register of Systematic Review (PROSPERO) (ID: CRD42022377065).

### 2.2. Ethics and dissemination

Because of the nature of the study, no ethical approval and no informed consent are required, and there will be no concerns about privacy. We will publish the findings and results of this research in a peer-reviewed journal.

### 2.3. Eligibility criteria

#### 2.3.1. Types of participants.

All studies including adult patients, aged 18 to 65 years and diagnosed with GAD by using any set of criteria, were eligible for inclusion, such as International Classification of Diseases, 10th Revision (ICD-10), Diagnostic and Statistical Manual of Mental Disorders Fifth Edition (DSM-5th) regardless of gender, educational background, nationality, or outpatient therapy or inpatient therapy.

#### 2.3.2. Type of interventions and comparators.

The study was divided into intervention group and control group. On the basis of the control group, the intervention group increased the use of clearly described complementary and alternative therapies, including herbal medicine, massage therapy, acupuncture, yoga, tai chi, meditation, qigong, mindfulness relaxation exercises and so on. The control group received conventional treatment or placebo. Other variables between groups should be strictly controlled.

#### 2.3.3. Types of outcomes.

*Primary outcomes*

The main outcome has to be measured by change score of a standardized anxiety rating scale (Hamilton Anxiety Rating Scale [HAMA]).^[22]^

*Secondary outcomes*

1. Response rates (≥50% decrease of baseline score in HAMA).^[23]^
2. Clinical efficiency.
3. Sleep quality standardized scale (the Pittsburgh Sleep Quality Index).^[24]^
4. Rates of adverse events.

#### 2.3.4. Types of studies.

This study will include all relevant randomized controlled trials (RCTs), quasi-RCTs will be excluded, such as using in clinic order allocation of RCT, case report is not included.

### 2.4. Search strategy

We will retrieve the following databases: the Chinese databases China National Knowledge Infrastructure, formerly Chinese Biomedical Database, Wanfang Data, Chongqing VIP Database for Chinese Technical Periodicals, the PubMed, MEDLINE, Embase, Web of Science and The Cochrane Database of Systematic Reviews, from their inception to January 1, 2023. We will search additional gray databases including OpenSIGLE, Greynet, etc. The language will be limited to English and Chinese. Take MEDLINE as an example, the detailed search strategy was presented in Table 1.

**Table 1:** Search strategy of the MEDLINE.

Number	Search terms
#1	Generalized Anxiety Disorder [Mesh]
#2	Generalized Anxiety Disorder [Title/Abstract] OR Generalized Anxiety Disorder [Title/Abstract] OR Anxiety Neuroses [Title/Abstract] OR Neuroses, Anxiety [Title/Abstract] OR Anxiety States, Neurotic [Title/Abstract] OR Neurotic Anxiety State [Title/Abstract]
#3	#1 OR #2
#4	Complementary and alternative therapies [Mesh]
#5	Complementary and alternative therapies [Title/Abstract] OR Complementary Therapies [Title/Abstract] OR Therapies, Complementary [Title/Abstract] OR Medicine, Complementary [Title/Abstract] OR Alternative Medicine [Title/Abstract] OR Acupuncture Therapy [Title/Abstract] OR herbal medicine [Title/Abstract] OR massage therapy [Title/Abstract] OR yoga [Title/Abstract] OR tai chi [Title/Abstract] OR meditation [Title/Abstract] OR mindfulness relaxation exercises [Title/Abstract]
#6	#4 OR #5
#7	randomized controlled trial [Title/Abstract] OR controlled clinical trial [Title/Abstract] OR clinical trial [Title/Abstract] OR randomized [Title/Abstract] OR RCT [Title/Abstract]
#12	#3 AND #6 AND #7

RCTs = randomized controlled trials.

### 2.5. Selection process of study

**Figure 1.**
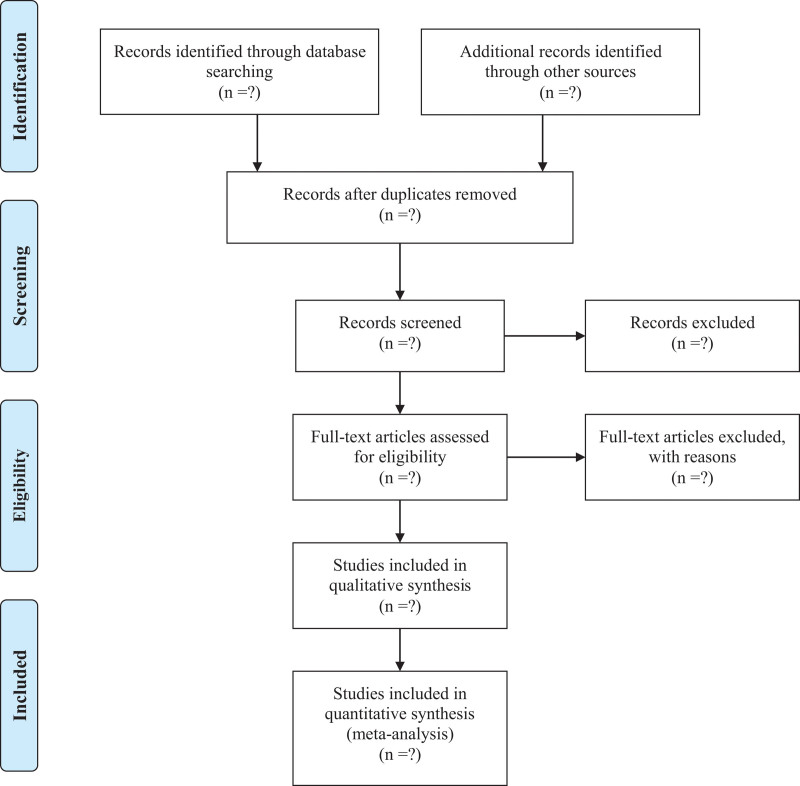
PRISMA flow diagram of study identification and selection. Preferred Reporting Items for Systematic Reviews and Meta-Analysis (PRISMA).

After an initial literature search, EndNote X9 software will be used for further literature selection and management, which will be done independently by 2 researchers (KS and YTW). Then the 3 researchers (YTW, LS, JWW) will read the full text and further screen the literature according to strict inclusion and exclusion criteria until they have a literature suitable for inclusion in the final NMA study. In this process, if there are any differences or inconsistencies between researchers, the corresponding author (RZ) will ultimately assist in the judgment. Figure 1 is a schematic diagram of literature selection in this study.

### 2.6. Data extraction and management

Two independent researchers (KS, YTW) will use a pre-designed data extraction table to extract information from the literature eventually included in the NMA. The data to be extracted includes: General information: title, journal, first author name, publication year, funding, random method; Participants information: sample size, gender, mean age, race, diagnostic criteria, disease course; Intervention information: type of therapy, clinical dosage, course of treatment; Outcomes: primary and secondary results, type of outcome measurement, adverse events.

### 2.7. Risk of bias assessment

Two researchers (LS, JWW) will use Cochrane Collaboration Risk of Bias Tool to assess the risk of bias, which included the 5 specific domains: randomization process, deviations from the intended interventions, missing outcome data, measurement of the outcome, selection of the reported outcome. If necessary, will be the third reviewer (RZ) to solve different opinions.

### 2.8. Data synthesis and statistical methods

#### 2.8.1. Selection of effect measure and effect model.

This study will use the SMD as the effect measure for continuous outcome variables, and use odds ratio (OR) to evaluate the effect size of dichotomous variables. Each effect amount will be expressed in a 95% confidence interval (CI).

#### 2.8.2. Assessment of heterogeneity.

The heterogeneity of the data in this study will be evaluated by calculating the *I*^2^ value. Heterogeneity will not be considered when *I*^2^ value is less than 50%. On the contrary, when *I*^2^ value is greater than 50%, we believe that there is a large heterogeneity, and we will further analyze the source of heterogeneity.

#### 2.8.3. Subgroup and sensitivity analyses.

If necessary, we will explore the possible sources of significant inconsistencies or heterogeneity through meta regression analysis and grouping. The subgroup analysis included age, different types of CAM treatments, duration of treatment and follow-up time.

#### 2.8.4. Network meta-analysis.

When the heterogeneity of the data is small, we believe that these studies have reasonable consistency and are suitable for further NMA. Before this, we will also use the node segmentation method to evaluate whether there is inconsistency between the direct evidence and indirect evidence of the same comparison. *P* > .05 indicates that there is good consistency, and it is suitable to continue to use NMA. We will conduct NMA through Stata V.15 and ADDIS software. Network element can compare multiple interventions at the same time, while retaining the internal randomization of individual trials. Therefore, we will use the NMA model for all studies within the Bayesian framework. Using a random effects NMA to analyze the primary and secondary outcomes, we will estimate all possible pairwise relative effects and rank the interventions for each CAM therapies. For each outcome, the distribution of ranking probabilities and surface under the cumulative ranking curve are used to estimate the relative rank of different CAM therapies. Report the results in SMD, along with 95% CI based on 100 000 Monte Carlo simulations and vague priors. The convergence of the model will be used to evaluate the stability of the analysis results.

#### 2.8.5. Publication bias.

If more than 10 studies are finally included, we will draw a funnel chart to assess publication bias. The Egger test was then used to assess the asymmetry of the funnel plot.

#### 2.8.6. Grading the quality of evidence.

This study will use the GRAD Eprofiler software to assess the quality of evidence. The quality of evidence will be divided into 4 levels: very low, low, moderate and high.

## 3. Discussion

At present, the Coronavirus disease 2019 (COVID-19) epidemic is spreading rapidly worldwide, and the number of confirmed cases and deaths continues to increase. The COVID-19 is a severe challenge to the global health system, not only posing a threat to people’s physical health, but also profoundly affecting people’s mental health. The people whose mental health is impaired include people infected with COVID-19, survivors and medical workers, and the reasons may include worries about personal safety, lack of effective treatment, isolation and blockade measures, unemployment risk, etc.^[25,26]^ GAD is one of the outstanding manifestations of people’s mental health problems during the COVID-19 pandemic. A web-based survey found that during the COVID-19 epidemic, the prevalence of anxiety worldwide was 35%.^[27]^ A survey by Galindo et al showed that 20.8% of 1508 participants had severe anxiety symptoms. Compared with other pandemics, there are more individuals with GAD in the context of COVID-19 epidemic, which needs attention and early intervention.^[28]^

CAM therapies have been proved to be a potential treatment for GAD. Especially in the context of COVID-19, the efficacy, safety and acceptability of CAM therapies for GAD deserve further study. Different from previous studies, this study focuses on discussing the differences between different CAM therapies, in order to guide clinical practice and provides reference for clinical decision-making.

There are some potential limitations are predictable to this study. For example, due to the limitation of language ability, the author only searched English and Chinese literature, which may lead to the potential risk of ignoring essential literature. In addition, difference of methodological quality in the trials may cause significant heterogeneity.

## Author contributions

KS was responsible for this study. KS, YTW and LS conceived and designed the study. LS, JWW and RZ participated in drafting the protocol and preparing the manuscript. All authors read and approved the final manuscript.

**Conceptualization:** Kai Song, Rong Zhang.

**Data curation:** Yating Wang, Li Shen, Jinwei Wang.

**Formal analysis:** Yating Wang, Li Shen, Jinwei Wang.

**Funding acquisition:** Kai Song.

**Investigation:** Rong Zhang.

**Methodology:** Kai Song, Yating Wang.

**Project administration:** Kai Song.

**Resources:** Kai Song.

**Software:** Li Shen, Jinwei Wang.

**Supervision:** Rong Zhang.

**Validation:** Yating Wang, Rong Zhang.

**Visualization:** Yating Wang, Li Shen, Rong Zhang.

**Writing – original draft:** Kai Song, Yating Wang, Li Shen, Jinwei Wang.

**Writing – review & editing:** Kai Song, Rong Zhang.
